# Mitochondrial Carriers and Substrates Transport Network: A Lesson from *Saccharomyces cerevisiae*

**DOI:** 10.3390/ijms22168496

**Published:** 2021-08-07

**Authors:** Alessandra Ferramosca, Vincenzo Zara

**Affiliations:** Department of Biological and Environmental Sciences and Technologies, University of Salento, I-73100 Lecce, Italy; vincenzo.zara@unisalento.it

**Keywords:** mitochondria, mitochondrial carrier, *Saccharomyces cerevisiae*, transport, metabolism

## Abstract

The yeast *Saccharomyces cerevisiae* is one of the most widely used model organisms for investigating various aspects of basic cellular functions that are conserved in human cells. This organism, as well as human cells, can modulate its metabolism in response to specific growth conditions, different environmental changes, and nutrient depletion. This adaptation results in a metabolic reprogramming of specific metabolic pathways. Mitochondrial carriers play a fundamental role in cellular metabolism, connecting mitochondrial with cytosolic reactions. By transporting substrates across the inner membrane of mitochondria, they contribute to many processes that are central to cellular function. The genome of *Saccharomyces cerevisiae* encodes 35 members of the mitochondrial carrier family, most of which have been functionally characterized. The aim of this review is to describe the role of the so far identified yeast mitochondrial carriers in cell metabolism, attempting to show the functional connections between substrates transport and specific metabolic pathways, such as oxidative phosphorylation, lipid metabolism, gluconeogenesis, and amino acids synthesis. Analysis of the literature reveals that these proteins transport substrates involved in the same metabolic pathway with a high degree of flexibility and coordination. The understanding of the role of mitochondrial carriers in yeast biology and metabolism could be useful for clarifying unexplored aspects related to the mitochondrial carrier network. Such knowledge will hopefully help in obtaining more insight into the molecular basis of human diseases.

## 1. Introduction

Mitochondria are subcellular organelles involved in different pathways. In addition to supplying energy, mitochondria contribute to many processes that are central to cellular function and that require the exchange of metabolites between the cytosol and the mitochondrial matrix [1].

These organelles are surrounded by a double-membrane system consisting of an outer mitochondrial membrane (OMM) that surrounds the inner membrane (IMM); the two membranes are separated by an intermembrane space. Numerous transport processes occur between the two mitochondrial membranes. The OMM contains large pores (porins), which are large enough to allow the passage of ions and molecules as large as a small protein [2]. The IMM is highly impermeable and, therefore, is characterized by the presence of specific carrier proteins which transport metabolites inside the mitochondria [3,4]. These proteins, which are encoded by nuclear DNA [5,6], play a fundamental role in cellular metabolism since they connect the intra-mitochondrial reactions with the extra-mitochondrial (cytosolic) ones.

The study of the structural properties of mitochondrial carrier proteins revealed common features, thus resulting in the concept of a family of mitochondrial transporters (mitochondrial carrier family or MCF) [7,8]. Therefore, all of them have (i) a length of about 300 amino acids; (ii) a tripartite structure consisting of three homologous domains, each made of about 100 amino acids; and (iii) a characteristic sequence motif PX(D/E)XX(K/R), known as “carrier signature”.

They all transport different substrates with common mechanisms, which are a strict counter-exchange of chemically related substrates (antiport), a unidirectional transport (uniport), or a substrate–proton transport (symport). This transport mechanism consists in the coordinated movement of six structural elements, which results in alternating opening and closing of the matrix or cytoplasmic side of the carriers. Each carrier has a single substrate-binding site and two gates. These gates are present on either side of the membrane and contain salt-bridge networks [8].

Despite similarity in their structure and transport mechanisms, mitochondrial carrier proteins are functionally diverse. The functional properties of the members of the MCF have been investigated in intact mitochondria and/or after reconstitution of the purified proteins into artificial membranes (liposomes) [9,10].

Mitochondrial carriers are widespread in all eukaryotes and considerable research has been conducted on characterizing the members of the MCF in yeast [11], mammals [4], plant [12], and insects [13]. In particular, in *Saccharomyces cerevisiae*, 35 members of the MCF have been identified and, in large part, functionally characterized [3,11]. With a single exception [14], these proteins are found in the inner membranes of mitochondria. By transporting several substrates across this membrane, they are indirectly involved in many biochemical processes, such as Krebs cycle, oxidative phosphorylation (OXPHOS), the transfer of reducing equivalents, gluconeogenesis, fatty acid metabolism, amino acid synthesis, and in cofactor transport.

The aim of this review is to describe the role of mitochondrial carriers so far identified in the yeast *Saccharomyces cerevisiae* in cell metabolism, attempting to critically analyze the functional connection between the substrates transported and the specific metabolic pathways to which they are addressed. We focused on *Saccharomyces cerevisiae* since this yeast can adapt its metabolism in response to specific growth conditions, different environmental changes, and nutrient depletion [15,16,17]. In fact, it preferentially uses glucose as a carbon source and fermentation is the major pathway for energy production, even under aerobic conditions. However, when glucose is unavailable, other carbon sources are used, requiring a shift to respiration [18]. This adaptation results in a metabolic reprogramming of genes and proteins involved in gluconeogenesis, glyoxylate cycle, and oxidative metabolism.

The metabolic adaptation, also known as metabolic flexibility, is essential for maintaining energy homeostasis also in human cells [19]. For example, metabolic reprogramming is a switch in metabolism that cells adopt in order to support an increase in their proliferative capacity or to undertake a specific differentiation pathway. In this context, yeast and some cancer cells share several features since, in both cells, glycolytic flux is stimulated concomitantly with the repression of OXPHOS, and fermentation is preferred, even under aerobic conditions [20].

Therefore, *Saccharomyces cerevisiae* can be used as a suitable model for gaining more insight into the pathogenic mechanisms of human diseases and the development of appropriate treatments [21,22,23].

## 2. Mitochondrial Carriers from *Saccharomyces cerevisiae*: An Overview

Before the sequencing of *Saccharomyces cerevisiae* genome, the functions of only five mitochondrial carriers were known: the three isoforms of the ADP/ATP carrier (Aac1p, Aac2p, and Aac3p) [24,25,26,27,28], the phosphate (Mir1p) [29], and the citrate carrier (Ctp1p) [30].

When the genomic sequence of *Saccharomyces cerevisiae* was completed in 1996, other mitochondrial carriers have been functionally characterized by a procedure based on the reconstitution of proteins expressed in *Escherichia coli* or in *Saccharomyces cerevisiae* [11]. These carriers include the dicarboxylate carrier (Dic1p) [31], the succinate/fumarate carrier (Sfc1p) [32], the ornithine carrier (Ort1p) [33], the carnitine carrier (Crc1p) [34], the oxaloacetate carrier (Oac1p) [35], the oxodicarboxylate carrier (Odc1/2p) [36], the aspartate/glutamate carrier (Agc1p, Ymc1/2p) [37,38], the S-adenosylmethionine carrier (Sam5p) [39], the thiamine pyrophosphate carrier (Tpc1p) [40], the GTP/GDP carrier (Ggc1p) [41], the pyrimidine nucleotide transporter (Pyt1p or Rim2p) [42], the carrier of NAD^+^ (Ndt1/2p) [43], the adenosine 5′-phosphosulfate carrier (Apsc1p) [44], and a novel carrier for citrate and oxoglutarate (Yhm2p or Coc1p) [45]. With this strategy, the yeast peroxisomal adenine nucleotide transporter was also identified and functionally reconstituted into liposomes (Ant1p) [14].

The functions of other additional yeast transporters have been identified by means of genetic studies, complementation of deletion strains, and measurements of transport activity in mitochondria or submitochondrial particles. These transporters include the carriers for ATP-Mg/ phosphate (Sal1p) [46], FAD (Flx1p) [47], Coenzyme A (CoA) (Leu5p) [48], iron (Mrs3/4p) [49,50], magnesium (Mme1) [51], pyridoxal 5’-phosphate (Mtm1p) [52], and the second isoform of the phosphate carrier (PiC2p) [53]. The different isoforms of mitochondrial carriers are expressed differently according to the metabolic state of the cell.

The other three members of the MCF from *Saccharomyces cerevisiae* are Ugo1p, which is a protein involved in the regulation of mitochondrial fusion [54]; the YDL119C gene product (Hem25), which is a glycine transporter [55], and the YFR045w gene product.

Ugo1p is classified as a member of MCF because it contains two putative carrier domains as well as the carrier signature. Distinct from the other mitochondrial carriers discussed here, this protein is inserted in the outer mitochondrial membrane, and it seems to be involved in the formation of mitochondrial fusion machinery, which plays a key role in the regulation of mitochondrial shape and number [54].

Finally, the presence of a possible transporter for pyruvate, which does not seem to belong to MCF, is still a debated matter of research [56,57]. This transport protein, known as MPC (Mitochondrial Pyruvate Carrier), is a non-canonical mitochondrial carrier because it does not possess the structural and functional properties of the members of MCF [56]. However, as canonical mitochondrial carrier proteins, MPC plays a central role in cellular homeostasis [57] since the transport of pyruvate into yeast mitochondria is an important step for oxidative energy metabolism as well as for the biosynthesis of organic compounds. In fact, in the mitochondrial matrix, pyruvate can be converted into acetyl-CoA by the pyruvate dehydrogenase complex, thus allowing the influx of carbon atoms into the Krebs cycle. Alternatively, pyruvate can be used as precursor for the synthesis of branched chain amino acids.

Table 1 reports the 35 members of the MCF identified in *Saccharomyces cerevisiae* as well as their functions, which include the transport of cofactors, nucleotides, and a variety of substrates. Therefore, the transport activities of these proteins need to be carefully orchestrated because it is very important to channel several molecules belonging to the same metabolic pathway in a fine and coordinated manner.

In the following paragraphs, we describe the involvement of the yeast mitochondrial carriers in specific metabolic pathways, such as OXPHOS, lipid metabolism, gluconeogenesis, and amino acids synthesis, which are reprogrammed depending on energy demand or supply. Interestingly, alterations or reprogramming of these pathways also represents a hallmark of several human diseases [58,59,60,61,62,63,64,65,66].

## 3. Mitochondrial Carriers and OXPHOS

*Saccharomyces cerevisiae* yeast cells may generate energy both by fermentation and aerobic respiration, depending on the type and availability of carbon sources. However, when glucose concentration is very low or when yeast cells grow on non-fermentable carbon sources, energy is produced by respiratory metabolism. Based on these characteristics, yeast is a powerful platform for gaining better understanding of the underlying molecular basis of human diseases [67]. In fact, although human mitochondria are more complex, the high similarity between yeast and human mitochondrial biogenesis and function renders *Saccharomyces cerevisiae* an excellent model for studying human mitochondrial physiopathology.

### 3.1. Transport of Substrates for ATP Synthesis

**Figure 1:**
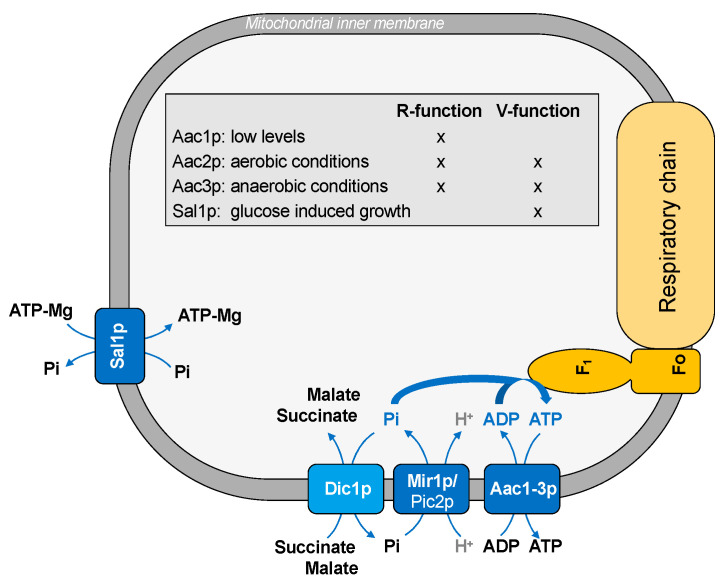
Transport of substrates into mitochondria for ATP synthesis.

Most cellular ATP is produced within the mitochondria by the biochemical pathway of OXPHOS. To this aim, ADP and Pi, which are required for ATP synthesis, are delivered across the inner membrane by specific mitochondrial carriers: the ADP/ATP carrier and the phosphate carrier (Figure 1).

The ADP/ATP carrier mediates the exchange of ADP into and ATP out of the mitochondrial matrix. Yeast has three isoforms of the ADP/ATP carrier, which are Aac1p, Aac2p, and Aac3p [24,25,26,27,28], and they are differently expressed according to the metabolic state of the cell. Aac1p is expressed at low levels, whereas Aac2p is the major isoform in yeast, which is expressed in aerobic growth conditions [27]; therefore, it is the only isoform required for respiration. Aac3p is expressed almost exclusively under anaerobic growing yeast [28]. Although the specific function of ADP/ATP carriers required for respiration is the exchange of cytosolic ADP for mitochondrial ATP, these carrier proteins may have a second function. This last function is the import of cytosolic ATP in mitochondria. According to this hypothesis, there is another ATP transporter, Sal1p, which is a carrier protein driving net ATP-Mg uptake into mitochondria. This protein catalyzes an electroneutral exchange of ATP-Mg (or free ADP) for Pi and is essential in the absence of Aac2p. Therefore, it has been suggested that ADP/ATP carriers have two different functions: R-function (ADP/ATP exchange required for respiratory growth) and V-function (ADP/ATP exchange required for viability). Aac1p, Aac2p, and Aac3p play a R-function; in addition, Aac2p and Aac3p, but not Aac1p, may also have a V-function, which is the only function belonging to Sal1p [46] (Figure 1). Thus, this last protein catalyzes a net uptake of ATP, which is responsible for the glucose-induced growth. A similar strategy is adopted by human cancer cells, where the isoform 2 of the ADP/ATP carrier (ANT2) imports glycolytically produced ATP into the mitochondria when OXPHOS is impaired [68]. Therefore, the presence of different isoforms of the ADP/ATP transporters suggest that the cellular ATP demand requires a strict coordination between different carrier proteins in terms of protein expression and/or direction of the transported substrates.

In order to synthesize ATP, inorganic phosphate needs to be transported into mitochondria. The mitochondrial phosphate carrier (Mir1p) catalyzes the proton co-transport of phosphate into the mitochondrial matrix. A minor form of phosphate carrier is Pic2p, which plays a role under specific stress conditions [53].

The functional interaction between ADP/ATP and phosphate carriers suggests that the transport of ADP and Pi across the inner mitochondrial membrane may be physically orchestrated, representing an example of the substrate channeling phenomenon. In fact, the transport of substrates belonging to the same metabolic pathway requires a high degree of flexibility and coordination. In this context, some authors [69] proposed a physical and spatial association between ADP/ATP and phosphate carriers; this association seems necessary for increasing the efficiency of the ATP synthase. Since the interaction of AAC1 and the phosphate carrier was demonstrated in human cells [70], it has been suggested that the mixed assembly of different mitochondrial carriers belonging to MCF (the so-called “carrier armada”) might be of general physiologic importance [69].

In this context, a key role may be played also by another carrier, which is the dicarboxylate carrier (Dic1p). This protein transports Pi outside mitochondria in exchange for Krebs cycle intermediates, such as succinate (which derives from cytosolic isocitrate by the action of isocitrate lyase, the first key enzyme of the glyoxylate cycle) or malate (which derives from cytosolic pyruvate by the action of pyruvate carboxylase and malate dehydrogenase) (Figure 1). Thus, given that phosphate is recycled back into the mitochondria by the phosphate carrier, the transport activity of these two carrier proteins requires strict coordination.

### 3.2. Transport of Reducing Equivalents

Redox homeostasis plays a key role to sustain metabolism and growth in all biological systems. The intracellular redox potential mainly depends on the NADH/NAD^+^ ratio and, to a lesser extent, by the NADPH/NADP^+^ ratio. Alteration in these ratios disturb physiological functions in humans, resulting in metabolic diseases, cancer, aging, and neurodegeneration disorders [71].

In *Saccharomyces cerevisiae*, the reduction of NAD^+^ to NADH occurs in several reactions. However, a metabolic compartmentation is guaranteed by the impermeability of the mitochondrial inner membrane for NADH and NAD^+^. The presence of these separate pools of cytosolic and mitochondrial NADH and NAD^+^ is not only relevant during aerobic respiratory growth but also during anaerobic fermentative growth.

In yeast, complex I of the mitochondrial respiratory chain is replaced by several peripheral membrane NADH dehydrogenases [72,73]. During respiratory growth, cytosolic and mitochondrial NADH is reoxidized by the respiratory chain, and the electrons are used for the reduction of quinone to quinol.

The existence of a functional malate-aspartate NADH shuttle in yeast was proposed with the identification of Agc1p, the counterpart of the human AGC (aspartate/glutamate carrier) [37]. Agc1p is a yeast mitochondrial aspartate/glutamate exchanger, which plays a role within the malate-aspartate NADH shuttle, necessary for growth on acetate and fatty acids as carbon sources. Unlike its human orthologues, this carrier also catalyzes glutamate uniport into mitochondria for nitrogen metabolism and ornithine synthesis [37].

**Figure 2:**
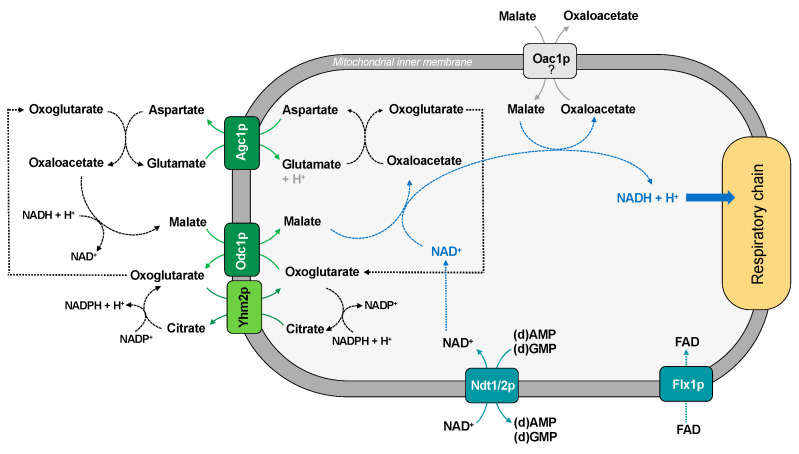
Transport of reducing equivalents into mitochondria.

The role of Agc1p in the malate-aspartate NADH shuttle needs to be coordinated with that of the oxodycarboxylate carrier (Odc1p), which is a carrier protein which transports intramitochondrial oxoglutarate in exchange for cytosolic malate [11] (Figure 2). Yeast has two isoforms of oxodycarboxylate carrier: Odc1p is the prevailing carrier isoform under respiratory conditions and is required for the yeast malate-aspartate shuttle, whereas Odc2p is the main isoform in the presence of glucose under anaerobic conditions [36].

Although there is no direct evidence of an exchange of malate and oxaloacetate by the oxaloacetate carrier (Oac1p), both intermediates are substrates of the carrier after its functional reconstitution into liposomes [35]. Therefore, we cannot exclude that they are transported in vivo. We can then hypothesize that, during respiratory growth, the malate/oxaloacetate shuttle could import NADH into the mitochondrial matrix (Figure 2); under anaerobic growth conditions, the shuttle could work in the reverse direction.

The mitochondrial NADH/NAD^+^ ratio also requires a strict coordination between NADH shuttle and the NAD^+^ transporter (Ndt1/2p) [74] (Figure 2).

Interestingly, it has been proposed that the NAD redox status is also able to regulate FAD homeostasis in *Saccharomyces cerevisiae* [75]. This suggests a parallel control of the translocation of FAD into mitochondria mediated by the FAD carrier (Flx1p). In fact, this last protein is much more than just a carrier protein since it is a regulator of succinate dehydrogenase level and flavination [76].

Finally, the citrate/oxoglutarate carrier (Yhm2p or Coc1p) is a carrier which catalyzes the citrate/oxoglutarate exchange and is involved in the cytosolic reduction of NADP^+^ that is required for biosynthetic and antioxidant reactions [45] (Figure 2). Moreover, there is no direct evidence of an exchange of substrates in the reverse direction under different metabolic conditions.

### 3.3. Transport of Krebs Cycle Intermediates

The Krebs cycle is tightly linked with OXPHOS. It produces reducing equivalents, such as NADH and FADH_2_, that deliver electrons to complexes of mitochondrial electron-transport chain. Moreover, Krebs cycle intermediates are also important molecules for biosynthetic purposes. Thus, Krebs cycle must be tightly regulated because its intermediates participate in both anabolism and catabolism depending on the energetic status of the cells. For this reason, new evidence confers a strategic role for Krebs cycle intermediates in the control of human physiology and disease [77]. According to this intriguing hypothesis, mitochondrial carriers could represent key points of metabolic regulation by connecting cytosolic and mitochondrial reactions.

**Figure 3:**
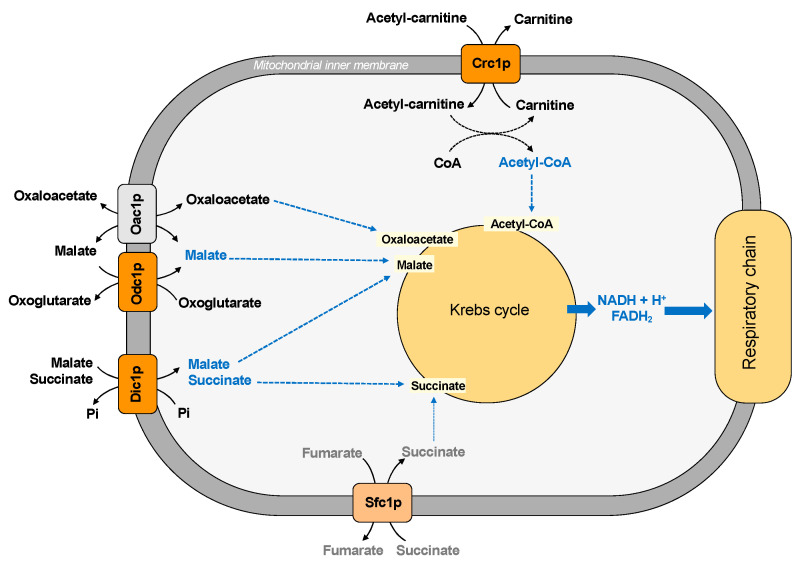
Mitochondrial carriers and transport of Krebs cycle intermediates.

*Saccharomyces cerevisiae* has two main pathways for replenishing the intermediates of the Krebs cycle: the glyoxylate cycle and the carboxylation of pyruvate to oxaloacetate. These pathways require the traffic of substrates across the inner mitochondrial membrane. In this context, the dicarboxylate carrier (Dic1p) supplies the Krebs cycle with cytoplasmic succinate (or malate) in exchange for phosphate, producing a net uptake of succinate [31] (Figure 3).

Succinate is a substrate also transported by another carrier protein, the succinate/fumarate carrier (Sfc1p), which exchanges external succinate for fumarate [32] (Figure 3). Therefore, the dicarboxylate and the succinate-fumarate carrier cooperates in connecting the synthesis of succinate in the cytosol to the mitochondrial succinate dehydrogenase, contributing to succinate homeostasis, which has been considered a new key regulator in cell biology [78]. In particular, it has been suggested that succinate/fumarate carrier does not play an anaplerotic role on acetate/ethanol and its main function seems to direct the carbon flux to synthetic pathways, such as gluconeogenesis [32] (see also paragraph five for further details). In agreement with this hypothesis, a lower activity of the Dic1p seems to favor the utilization of cytosolic succinate in the gluconeogenic pathway [11].

Other Krebs cycle intermediates are malate and oxaloacetate. The exchange between intramitochondrial oxoglutarate and cytosolic malate, which is also required for the malate/aspartate shuttle (see Section 3.2), supplies the Krebs cycle with malate and requires the transport activity of the oxodicarboxylate carrier (Odc1p) (Figure 3).

The mitochondrial uptake of oxaloacetate is guaranteed by the presence of the oxaloacetate carrier (Oac1p) [35] because yeast mitochondria do not possess the enzyme pyruvate carboxylase, which is necessary to produce oxaloacetate. In fact, in contrast to mammalian pyruvate carboxylase, which is localized in the mitochondrial matrix, the two yeast isoforms of this enzyme are cytosolic [78].

Moreover, Crc1p supplies the first substrate of Krebs cycle since it transports Acetyl-CoA deriving from fatty acid oxidation in the mitochondrial matrix in exchange for carnitine [34].

## 4. Mitochondrial Carriers and Lipid Metabolism

Over the last decades, yeast has become a key model organism for the study of lipid biochemistry, playing a key role not only in biotechnological applications [79] but also in the study of human diseases and of cancer [80,81,82]. In fact, metabolic fluxes observed in cancer cells appear very similar to those observed in the fermenting and rapidly proliferating yeast. However, yeast cells show a moderate complexity in comparison to mammalian cells.

**Figure 4:**
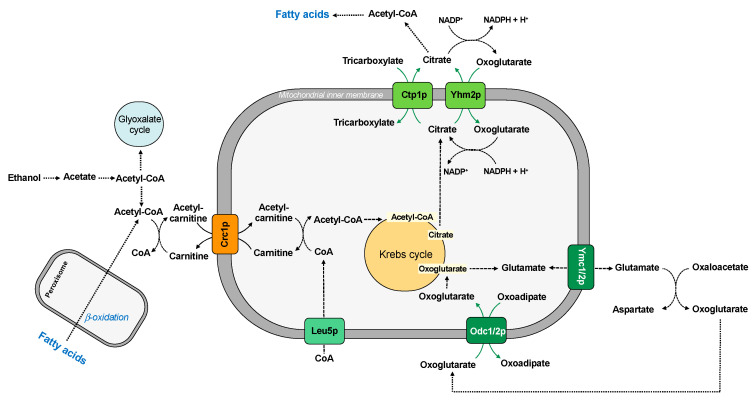
Mitochondrial carriers and lipid metabolism.

The utilization of fatty acids by *Saccharomyces cerevisiae* requires coordinated function of peroxisomes and mitochondria. In peroxisomes, fatty acids are oxidized to acetyl-CoA, which is converted to acetyl-carnitine. This molecule is imported into the mitochondria by Crc1p for its further metabolization in exchange for free carnitine [34]. This carrier protein may also participate in the utilization of ethanol, which is converted in the cytosol to acetate and then activated to acetyl-CoA. The acetyl group can be metabolized by the glyoxylate pathway or transferred to carnitine in the form of acetyl-carnitine and then transported to mitochondria (Figure 4). The pool of CoA in the mitochondrial matrix is also guaranteed by the CoA carrier (Leu5p) [48].

In the mitochondrial matrix, the acetyl group is transferred to oxaloacetate to form citrate and to begin the Krebs cycle.

In yeast, the presence of oleic acid as a sole carbon source induces the expression of enzymes and proteins involved in the fatty acid oxidation. In this condition, *Saccharomyces cerevisiae* requires the transport activity of the two mitochondrial oxodicarboxylate transporters, Odc1p and Odc2p, for its efficient growth, which catalyze the counter-exchange of oxoglutarate/oxoadipate. In fact, the oxoglutarate transported by the carrier is used to synthesize glutamate, and the glutamate-sensitive retrograde signaling pathway is important for upregulation of peroxisomal function where fatty acid oxidation occurs [83] (Figure 4). It has been also proposed that two additional mitochondrial transporter family members, the isoforms of aspartate/glutamate carrier Ymc1p and Ymc2p, are involved in glutamate metabolism [37,38], confirming an indirect connection between fatty acid β-oxidation and glutamate biosynthesis. Since these four transporters have overlapping activity in oleic acid utilization and glutamate homeostasis, yeast must express at least one of them for growth on oleic acid.

It is important to underline that the yeast *Saccharomyces cerevisiae* does not typically feed on fatty acids and carnitine shuttle requires supplementation of carnitine to the medium as the yeast is not able to synthesize this metabolite. Thus, cellular function and yeast growth relies mainly on endogenous fatty acid synthesis [84]. Since these metabolic fluxes are very similar to those observed in cancer cell physiology, understanding lipid metabolism in *Saccharomyces cerevisiae* may contribute to understanding fundamental regulatory mechanisms of lipid synthesis in cancer [80].

To this regard, citrate is a key intermediate in both carbohydrate and lipid metabolism and is formed in the first reaction of Krebs cycle. Citrate can be processed in the Krebs cycle to generate reducing equivalents, thus resulting in the production of ATP. It can also be transported to the cytosol across the inner membrane [30]. The citrate can be transported to cytosol by the citrate carrier (Ctp1p) in exchange for another tricarboxylate (citrate or isocitrate) or by the citrate/oxoglutarate carrier (Yhm2p). This molecule serves as the major acetyl-CoA supply for fatty acid synthesis, as suggested by comparative study of citrate efflux [85,86] (Figure 4).

Cytosolic citrate could be also converted to oxoglutarate by cytosolic isocitrate dehydrogenase and, during this reaction, NADP^+^ is converted to NADPH, thus increasing the NADPH reducing power in the cytosol [45].

Therefore, in yeast cells there are two carrier proteins involved in the transport of citrate in the cytosol. In contrast to the corresponding isoform of citrate transport protein characterized in higher eukaryotic cells [87,88,89,90], very little is known about the yeast mitochondrial citrate transporter Ctp1p, which shows a strict substrate selectivity for citrate or isocitrate; malate is an alternative substrate, but it can be transported to a considerably lesser extent [30]. On the other hand, the citrate/oxoglutarate carrier Yhm2p also plays a key role in the NADPH redox shuttle, contributing to biosynthetic and antioxidant reactions.

## 5. Mitochondrial Carriers and Gluconeogenesis

Gluconeogenesis is a regulated pathway, which is usually switched on in times of carbohydrate deficiency. In particular, the expression of gluconeogenic genes is coregulated with that of many respiratory genes because OXPHOS is required to obtain energy necessary during gluconeogenic processes.

*Saccharomyces cerevisiae* is an excellent model organism for the study of gluconeogenesis and its regulation. In fact, yeast can highly induce gluconeogenesis when non-fermentable carbon sources (such as ethanol or acetate) must be utilized; this metabolic pathway can be downregulated in the presence of carbohydrates.

**Figure 5:**
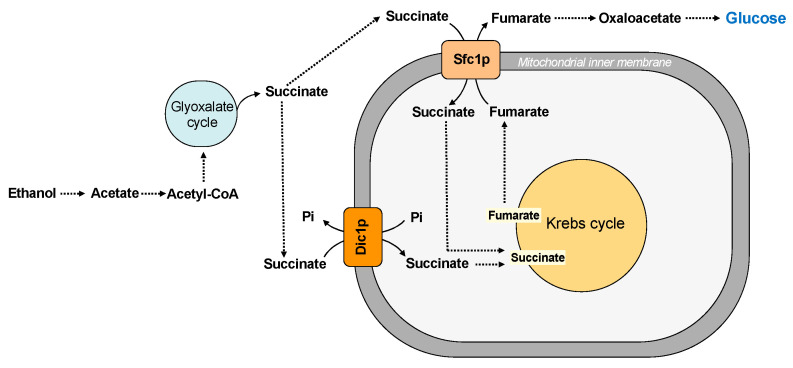
Mitochondrial carriers and gluconeogenesis.

The succinate/fumarate carrier (Sfc1p) is a carrier protein involved in gluconeogenesis from acetate and ethanol since it transports cytoplasmic succinate into the mitochondrial matrix in exchange for fumarate. In the cytosol, fumarate can be converted first to malate and then to oxaloacetate, which enters gluconeogenesis [32] (Figure 5). Therefore, when sufficient levels of Krebs cycle intermediates are provided by the dicarboxylate carrier (Dic1p) in the presence of ethanol and acetate, *Saccharomyces cerevisiae* directs the carbon flux to synthetic pathways via the succinate/fumarate carrier (Sfc1p). In fact, Dic1p is not required for gluconeogenesis from pyruvate, lactate, and glycerol; thus, its primary function is to transport cytoplasmic dicarboxylates into the mitochondrial matrix rather than to direct carbon flux to gluconeogenesis (Figure 5). However, a possible involvement of this carrier protein in gluconeogenesis from ethanol or acetate by exporting malate from mitochondria is a controversial matter [91].

## 6. Mitochondrial Carriers and Amino Acids Synthesis

Amino acids are essential for cell growth and proliferation because they are used for protein, nucleotide, and fat biosynthesis but they are also degraded to produce energy.

Mitochondria play a key role in amino acid homeostasis according to the demand and resources of a cell [92]. In this context, the transport activity of mitochondrial carriers needs to be coordinated to transport intermediates involved in anabolic and catabolic processes across the inner mitochondrial membrane [93].

In this paragraph, we describe the role of some mitochondrial carriers involved in amino acid synthesis. Interestingly, these proteins are also involved in other metabolic pathways, suggesting that mitochondrial carrier proteins play multiple metabolic roles in yeast metabolism.

The yeast *Saccharomyces cerevisiae* is perhaps the most suitable model organism for studying the link between specific components of the diet, such as amino acids, and cellular protection and aging. For example, arginine could be involved in the reduction in cellular oxidative stress as well as in the protection from damage caused by ethanol stress. Based on this evidence, arginine synthesis pathway is involved in cell protection from oxidative damage [94].

**Figure 6:**
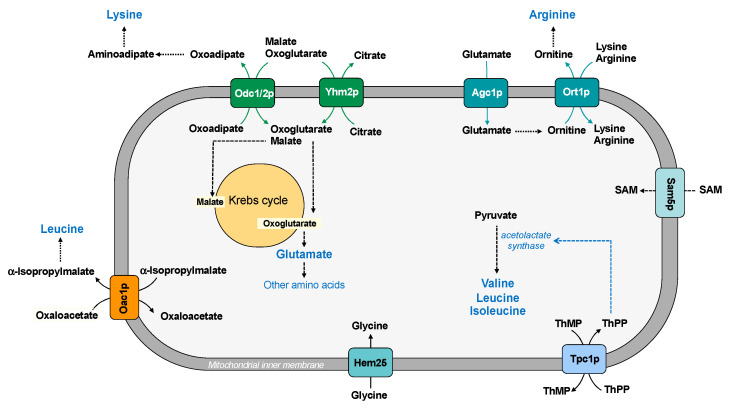
Mitochondrial carriers and amino acid synthesis. ThPP: thiamine pyrophosphate; ThMP: thiamine monophosphate; SAM: S-adenosylmethionine.

The biosynthesis of arginine requires the participation of mitochondrial and cytosolic reactions. In fact, glutamate must be translocated from the cytosol to the mitochondrial matrix where it is converted to ornithine. It has been suggested that the aspartate/glutamate carrier (Agc1p) is involved in the transport of glutamate into the mitochondria for nitrogen metabolism and ornithine synthesis. In order to perform this function, Agc1 acts as uniporter, whereas the same carrier protein acts as antiporter of aspartate/glutamate in the malate-aspartate NADH shuttle [37]. Ornithine is transported out of mitochondria by the ornithine carrier (Ort1p), where it is converted to arginine. Moreover, arginine can pass the mitochondrial membrane by using the same carrier, which also transports lysine (Figure 6).

Three mitochondrial transporters are essential for lysine biosynthesis [95]. In *Saccharomyces cerevisiae*, the synthesis of lysine occurs in the cytosol, where oxoadipate, which is produced in the mitochondrial matrix, is transaminated to aminoadipate and then in lysine. Since the oxodicarboxylate carrier (Odc1/2p) catalyzes an antiport reaction, the efflux of oxoadipate requires the uptake of oxoglutarate, malate, or another Krebs cycle intermediate (according to the metabolic conditions) [36] (Figure 6). It is important to underline that Odc1p is the main isoform under respiratory conditions, whereas Odc2p is the major isoform in the presence of glucose; the citrate/oxoglutarate carrier (Yhm2p) is present on both fermentable and nonfermentable carbon sources. Thus, at least one isoform of Odc and Yhm2p is present in different growth conditions to assure the synthesis of lysine. In addition, the citrate/oxoglutarate carrier Yhm2p is essential for increasing the NADPH reducing power in the cytosol (see paragraph 4).

The oxaloacetate carrier (Oac1p) is a carrier involved in leucine biosynthesis because it transports α-isopropylmalate, produced in the mitochondrial matrix, to the cytosol in exchange for oxaloacetate. According to this transport function, in yeast strains lacking this carrier protein, leucine supplementation is required for optimal growth on fermentable carbon sources [96] (Figure 6).

Mitochondria are the major site of branched chain amino acids biosynthesis [97]. In fact, in *Saccharomyces cerevisiae*, the entire pathway for synthesis of isoleucine and valine is localized in mitochondria, whereas the pathway for synthesis of leucine is localized both in the mitochondria and cytosol [98].

The acetolactate synthase, which catalyzes the first step in the synthesis of the branched chain amino acids, is a thiamine pyrophosphate (ThPP)-dependent enzyme. Tpc1p is a carrier protein involved in supplying the cofactor ThPP required for enzyme functioning [40] (Figure 6). Therefore, mitochondrial carriers are involved in specific metabolic pathways also transporting cofactor of several important enzymes.

In addition to the described mitochondrial transporters, MCF includes two other members that transport amino acids: Sam5p and Hem25 (YDL119c product) [55] (Figure 6). Sam5p transports into mitochondria S-adenosylmethionine, which is used as donor of a methyl group for methylation reactions and for biotin and lipoic acid synthesis [39]. Hem25 has been proposed as a transporter of glycine into the mitochondria, where it is used for heme biosynthesis [55]. Mitochondrial carriers that transport serine or proline have not been found [98]. However, yeast cells in the presence of appropriate sources of carbon and ammonium are able to synthesize all amino acids. Therefore, families of amino acids can derive from a common molecule, such as glutamate, and only few mitochondrial transporters are required to assure adequate amino acid levels in the mitochondrial matrix for cellular processes.

## 7. Conclusions

Despite the considerable progress made in the last years in characterizing mitochondrial carriers from *Saccharomyces cerevisiae*, many aspects related to their role in metabolic pathways connecting mitochondrial and cytosolic reactions remain to be ascertained. In fact, the function of most transport proteins has been determined by expressing the gene in *Escherichia coli* or *Saccharomyces cerevisiae,* reconstituting protein into liposomes and testing its transport activity in the presence of substrates selected on the basis of genetic, biochemical, and phylogenetic considerations [11].

However, an intriguing aspect is the correlation between the in vitro activities of characterized carriers and their specific physiological roles in vivo.

In this review, we tried to match the results obtained by in vitro studies, associating different carrier proteins to specific biochemical processes. We are aware that our analysis provides only a partial view of the complex network of mitochondrial carrier proteins and metabolic pathways in which they are involved. However, we believe that the analysis proposed would be useful for stimulating further experiments in order to elucidate the full picture of metabolic interrelationships and of carrier proteins crosstalk in yeast.

Our overview revealed that all carrier proteins which are involved in the same metabolic pathway should be orchestrated to adequately channel substrates toward the formation of desired products. Interestingly, the same mitochondrial carrier can transport different substrates (as suggested by experiments of substrate specificity in reconstituted liposomes) depending on the metabolic pathway in which it is involved.

Taken together, all this evidence suggests that mitochondrial carriers play a key role in the adaptation of yeast metabolism in response to specific growth conditions. Their involvement in metabolic pathways can provide new insights not only on the physiological roles of mitochondrial carriers in yeast cell metabolism but also in the understanding of molecular basis of human diseases.

However, many questions remain to be addressed. In fact, some carrier proteins possess specific isoforms which possess different physiological roles. At the same time, different carrier proteins can transport the same substrates depending on metabolic condition of the cell. What, when, and how much are these proteins expressed?

Are these proteins distributed in the mitochondrial inner membrane, or are they localized to create specific carrier clusters associated to specific metabolic pathways?

Is there a physical interaction between carrier proteins contributing to the same biochemical process?

How are these proteins orchestrated to channel substrates to common pathways?

These unexplored aspects are very intriguing, and more research is required to highlight new findings in the field of mitochondrial carrier biology in the yeast *Saccharomyces cerevisiae*.

## Figures and Tables

**Table 1:** List of mitochondrial carriers from *Saccharomyces cerevisiae*. Alternative carrier names are in brackets.

Carrier		Substrates Transported	Function/Metabolic Pathway
ADP/ATP carrier	Aac1p	ADP, ATP	Oxidative phosphorylation
	Aac2p		
	Aac3p		
ADP/ATP carrier (peroxisomal)	Ant1p	ATP, AMP	Lipid metabolism
Adenosine 5′-phosphosulfate carrier	Apsc1p	Adenosine 5’-phosphosulfate 3’-phospho-adenosine 5’-phosphosulfate, sulfate, and phosphate	Thermotolerance and synthesis of methionine and glutathione at elevated temperatures
ATP-Mg/phosphate carrier	Sal1p	ADP, ATP, ATP-Mg, and Pi (Ca^2+^-stimulated)	Glucose-induced calcium signal
Aspartate/glutamate carrier	Agc1p	Aspartate, glutamate	Nitrogen metabolism and ornithine synthesis Malate-aspartate NADH shuttle
	Ymc1p Ymc2p		
Carnitine carrier	Crc1p	Carnitine, acetyl-carnitine, and propionyl-carnitine (medium- and long-chain acyl-carnitines less efficiently)	Lipid metabolism
Citrate carrier	Ctp1p	Citrate, tricarboxylates	Lipid and glucose metabolism
Citrate/oxoglutarate carrier	Yhm2p (Coc1p)	Citrate, oxoglutarate (oxaloacetate, succinate, and fumarate less efficiently)	Increase in the NADPH reducing power in the cytosol Component of the citrate-oxoglutarate NADPH redox shuttle
Coenzyme A carrier	Leu5p	Coenzyme A	Distribution of Coenzyme A
Dicarboxylate carrier	Dic1p	Dicarboxylates (malate, succinate, or malonate), Pi, sulfate, and thiosulfate	Anaplerotic role for the Krebs cycle
FAD carrier	Flx1p	FAD	Flavin transport
GTP/GDP carrier	Ggc1p	GTP, GDP, dGTP, dGDP, and the structurally related ITP and IDP (guanosine 5′-tetraphosphate and the (deoxy)nucleoside di- and triphosphates of U and T less efficiently)	Protein synthesis and RNA synthesis
Magnesium carrier	Mme1	Magnesium	Homeostasis of magnesium
NAD^+^ carrier	Ndt1p	NAD^+^ (dAMP and dGMP, NADH, NADP^+^, or NADPH less efficiently)	Import NAD^+^ into mitochondria
	Ndt2p		
Iron carrier	Mrs3p	Iron	Iron accumulation
	Mrs4p		
Ornithine carrier	Ort1p	Ornithine/H^+^ or ornithine/ornithine (arginine and lysine less efficiently)	Arginine synthesis
Oxaloacetate carrier	Oac1p	Oxaloacetate, sulfate, and α-isopropylmalate (various substrates of the dicarboxylate and oxoglutarate carriers less efficiently)	Anaplerotic role for the Krebs cycle Leucine synthesis
Oxodicarboxylate carrier	Odc1p	Oxoadipate, oxoglutarate (dicarboxylates and malate less efficiently)	Nitrogen assimilation Malate/aspartate shuttle
	Odc2p		
Phosphate carrier	Mir1p	Phosphate	Oxidative phosphorylation
	Pic2p		
Pyridoxal 5’-phosphate transporter	Mtm1p	Pyridoxal 5’-phosphate transporter	Pyridoxal 5’-phosphate trafficking Iron homeostasis
Pyrimidine nucleotide carrier	Pyt1p (Rim2p)	Pyrimidine (deoxy)nucleoside mono-, di- and triphosphates	mtDNA and mtRNA synthesis
S-adenosylmethionine carrier	Sam5p	S-adenosylmethionine	Biosynthesis of biotin and lipoic acid Methylation reactions of mtDNA, mtRNA, and mitochondrial proteins
Succinate/fumarate carrier	Sfc1p	Succinate, fumarate	Gluconeogenesis
Thiamine pyrophosphate carrier	Tpc1p	Thiamine pyrophosphate, thiamine monophosphate ((deoxy)nucleotides less efficiently)	Branched chain amino acids synthesis
	Ugo1p		Mitochondrial fusion
	YDL119c (Hem25)	Glicine	Heme synthesis
	YFR045w product	?	?
